# Balanced chaos: a 55-year journey with unrepaired D-transposition of great arteries, ventricular septal defect, and subvalvular and valvular pulmonary stenosis

**DOI:** 10.1093/ehjcr/ytae257

**Published:** 2024-05-17

**Authors:** Samah El-Mhadi, Hind Hibatouallah, Zakia Touati, Mohammed Cherti

**Affiliations:** Cardiology B Department, Ibn Sina University Hospital Center, Mohammed V University, Rabat, Morocco; Cardiology B Department, Ibn Sina University Hospital Center, Mohammed V University, Rabat, Morocco; Cardiology B Department, Ibn Sina University Hospital Center, Mohammed V University, Rabat, Morocco; Cardiology B Department, Ibn Sina University Hospital Center, Mohammed V University, Rabat, Morocco

We report a case of a 55-year-old homeless patient with congenital heart disease (CHD) who lacked prior cardiac monitoring due to his precarious lifestyle.

He presented with worsening exertional dyspnoea, and on admission, had a blood pressure of 130/80 mmHg and heart rate of 70 b.p.m. Physical examination revealed digital clubbing, generalized cyanosis, and peripheral oxygen saturation of 85%. A holosystolic murmur was detected in the precordial area. ECG demonstrated sinus rhythm at 70 b.p.m. with evidence of bi-atrial and bi-ventricular hypertrophy.

**Figure 1:**
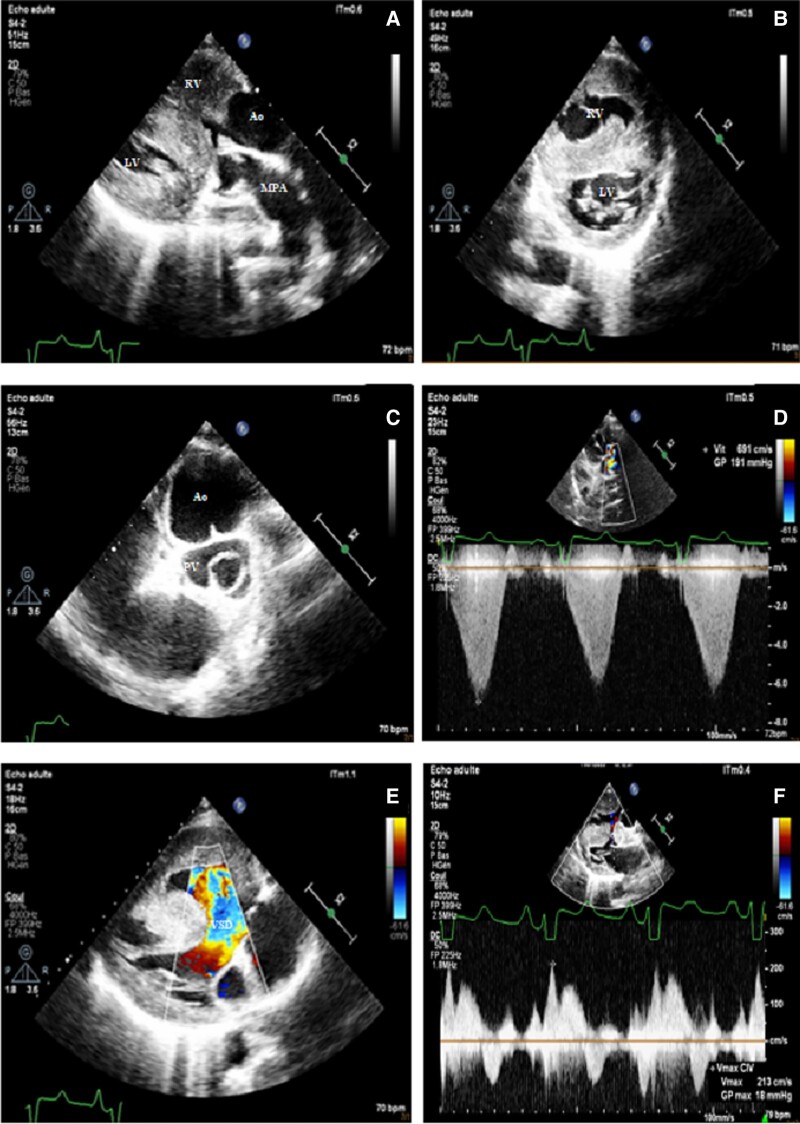
(*A*) TTE parasternal long-axis view demonstrating D-transposition of the great arteries, with the aorta (Ao) originating from the right ventricle (RV) and the main pulmonary artery (MPA) arising from the left ventricle (LV). (*B*) TTE parasternal short-axis view revealing right ventricular (RV) hypertrophy with flattening of the septal curvature in systole. (*C*) TTE parasternal short-axis view showing the tricuspid and anterior-right position of the aortic valve (Ao), and the bicuspid and posterior-left position of the pulmonary valve (PV). (*D*) TTE modified parasternal long-axis view showing multi-level pulmonary obstruction due to sub-pulmonary conus and bicuspid pulmonary valve, with maximum outflow tract velocity at 6.9 m/s. (*E*) TTE parasternal long-axis view demonstrating a large and non-restrictive outlet ventricular septal defect (VSD) causing right-to-left shunting in diastole. (*F*) TTE parasternal long-axis view showing a large and non-restrictive outlet VSD causing left-to-right shunting in systole (max velocity: 2.1 m/s).

Transthoracic echocardiography revealed D-transposition of the great arteries (d-TGA) (*Figure 1A*), with large and non-restrictive outlet ventricular septal defect causing left-to-right shunting in systole and right-to-left shunting in diastole (*Figure 1E* and *F*). Multi-level pulmonary obstruction was observed due to sub-pulmonary conus and a bicuspid pulmonary valve (*Figure 1C* and *D*), leading to hypertrophied right ventricle (*Figure 1B*).

Laboratory results indicated polycythaemia (haemoglobin: 22 g/dL), iron deficiency (ferritin: 57 mg/L), and hyperuricaemia at 160 mg/L with normal renal function.

Due to the delayed age of diagnosis, limited literature supporting long-term outcomes after d-TGA surgery in adulthood, the patient’s socioeconomic status, and limited access to specialized unit care for adults with CHD, right heart catheterization and surgical repair were not considered.

Palliative care approach was implemented (intravenous hydration, phlebotomies, iron supplementation, allopurinol, and preventive measures against infective endocarditis).

D-transposition of the great arteries is a rare and potentially fatal CHD, accounting for 5% of all congenital heart defects, with a 90% mortality rate within the first year of life.^1^ The presentation of unrepaired d-TGA in adulthood is exceedingly rare. The longest reported life expectancy of unoperated TGA was observed in a 40-year-old female.^2^ This case highlights the challenges faced by adults with complex CHD and vulnerable social contexts, emphasizing the importance of early diagnosis, tailored care plans, and comprehensive support structures to optimize outcomes.^3^
